# Membrane lipids and maximum lifespan in clownfish

**DOI:** 10.1007/s10695-021-01037-1

**Published:** 2021-12-04

**Authors:** Pedro F. Almaida-Pagan, Alejandro Lucas-Sanchez, Antonio Martinez-Nicolas, Eva Terzibasi, Maria Angeles Rol de Lama, Alessandro Cellerino, Pilar Mendiola, Jorge de Costa

**Affiliations:** 1 Chronobiology Lab, Department of Physiology, Faculty of Biology, University of Murcia, Mare Nostrum Campus, IUIE, IMIB-Arrixaca, 30100 Murcia, Spain; 2 Ciber Fragilidad y Envejecimiento Saludable (CIBERFES), Madrid, Spain; 3 Stazione Zoologica Anton Dohrn, Naples, Campania Italy; 4 Department of Neurosciences, Bio@SNS, Scuola Normale Superiore, Pisa, Italy

**Keywords:** Membranes, Lifespan, Fish, Lipids, Peroxidation

## Abstract

The longevity-homeoviscous adaptation (LHA) theory of ageing states that lipid composition of cell membranes is linked to metabolic rate and lifespan, which has been widely shown in mammals and birds but not sufficiently in fish. In this study, two species of the genus *Amphiprion* (*Amphiprion percula* and *Amphiprion clarkii*, with estimated maximum lifespan potentials [MLSP] of 30 and 9–16 years, respectively) and the damselfish *Chromis viridis* (estimated MLSP of 1–2 years) were chosen to test the LHA theory of ageing in a potential model of exceptional longevity. Brain, livers and samples of skeletal muscle were collected for lipid analyses and integral part in the computation of membrane peroxidation indexes (PIn) from phospholipid (PL) fractions and PL fatty acid composition. When only the two *Amphiprion* species were compared, results pointed to the existence of a negative correlation between membrane PIn value and maximum lifespan, well in line with the predictions from the LHA theory of ageing. Nevertheless, contradictory data were obtained when the two *Amphiprion* species were compared to the shorter-lived *C. viridis*. These results along with those obtained in previous studies on fish denote that the magnitude (and sometimes the direction) of the differences observed in membrane lipid composition and peroxidation index with MLSP cannot explain alone the diversity in longevity found among fishes.

**Supplementary Information:**

The online version contains supplementary material available at 10.1007/s10695-021-01037-1.

## Introduction

Reactive oxygen species (ROS) constitute the only known molecules endogenously and continuously produced by cells that have the capacity to break covalent bonds, causing damage to tissue macromolecules in biological systems (Barja 2019). Scientific evidence continues to support the mitochondrial oxygen free radical theory of ageing (Barja 2013, 2019; Miwa et al. 2014; Shen et al. 2014; López-Lluch et al. 2015; Zsurka et al. 2018) both between and within animal species. Short-lived mammals and birds have species-specific high mitochondrial ROS production (mitROSp) rates at complex I of the electron transport chain (Ku et al. 1993; Barja and Herrero 1998; Herrero and Barja 1998; Barja 2004; Lambert et al. 2007; Csiszar et al. 2012). Although ROS damage affects all cell macromolecules, lipid peroxidation is quantitatively the main oxidative process in tissues due to the high sensitivity to oxidation of polyunsaturated fatty acids (PUFA), which are essential constituents of cell membrane phospholipids (PL) (Bielski et al. 1983). Besides, lipid peroxidation is an exponential reaction chain process that generates many toxic and mutagenic by-products like the aldehydes hydroxynonenal or malondialdehyde, which can diffuse throughout the cell including the nucleus, which is poor in lipids (Chaudhary et al. 1994). Reaching the nucleus by diffusion, those aldehydes chemically react with free amino groups in DNA and could contribute to DNA damage, both in the nucleus and mitochondria.

The longevity-homeovicous adaptation (LHA) theory of ageing states that lipid composition of cell membranes (particularly that of mitochondria) is linked to metabolic rate and lifespan, which has been shown in a wide number of animal species (Pamplona et al. 1998; 2000). In comparative studies, performed on various species of mammals and birds, it has been found that species with a shorter lifespan have more unsaturated membranes than species with a longer lifespan (Pamplona 2008). Membranes with high levels of PUFA are more fluid, and the LHA theory of ageing suggests that this can enable or promote higher molecular activity of membrane proteins and, in turn, increase the metabolic activity of cells, tissues and, consequently, whole animals. At the same time, susceptibility to oxidative damage increases with the proportion of PUFA in membranes (Pamplona et al. 1998). In order to test the LHA theory of ageing in fish, where very little information is available (see Hulbert et al. 2007), we recently published a study on fishes of genus *Nothobranchius* (de Costa et al. 2020), which includes some of the shortest-lived vertebrates in nature (3–18 months, depending on the species) and has proved to be a remarkable system for gerontological research (Lucas-Sanchez et al. 2014; Tozzini et al. 2013). In these fishes, the longer-lived fish species have more saturated membranes and, therefore, a lower susceptibility to oxidative damage, as the LHA theory posits (de Costa et al. 2020).

On the other hand, clownfish of genus *Amphiprion* have been proposed as the first experimental models for long-lived vertebrates as some of its species have been reported by hobbyists (in captivity) and by researchers (in the wild) to live for more than two decades (Sahm et al. 2019). These fishes evolved a specific adaptation that allows them to live in symbiosis with sea anemones (Buston and García 2007). Under the anemone’s protection, the extrinsic mortality rate of these fish is low, and, following the standard evolutionary theories of ageing, low extrinsic mortality conditions lead to the evolution of slow senescence and increased lifespan (Mariscal 1970; Aldenhoven 1986; Eckert 1987; Elliot et al. 1995; Buston 2003; Blanco and Sherman 2005).

In this study, two species of the genus *Amphiprion* (*Amphiprion percula* and *Amphiprion clarkii*, with estimated maximum lifespan potentials [MLSP] in the wild of 30 and 9–16 years, respectively) (Moyer 1986; Buston and García 2007; Sahm et al. 2019) were studied along with the damselfish *Chromis viridis*, to test how the LHA theory of ageing applies to this fish group. *C. viridis* belong to the non-symbiotic sister-taxon of the *Amphiprion* genus, share with them general traits linked to their life in nature and show an interesting relationship with branching corals (Garcia-Herrera et al. 2017). However, despite the presence of a favourable microhabitat, *C. viridis* are predated by a wide range of generalist predator species, which has been suggested to be linked to a higher mortality rate (Hixon and Carr 1997; Sahm et al. 2019) and shorter lifespan (estimated MLSP of 1–2 years) (Wantiez and Thollot 2001; Sahm et al. 2019).

## Methods

### Animal housing and sampling

**Fig. 1:**
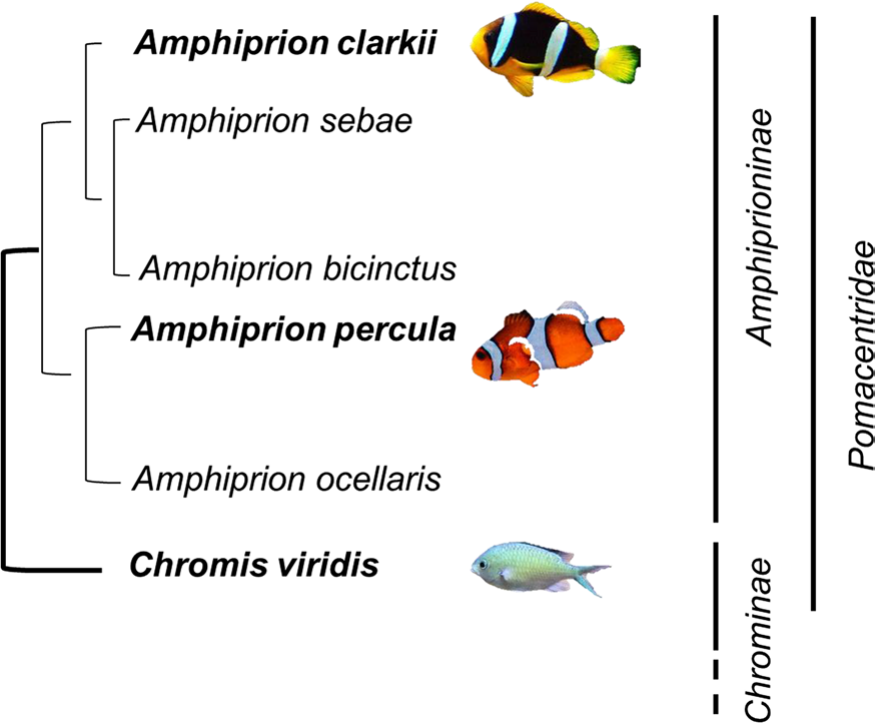
Nucleotide-based phylogeny of the analysed fish species as estimated in a previous study (Sahm et al. 2019). *Chromis viridis* represents the non-symbiotic sister-taxon of the *Amphiprion* genus. *A. percula* photo by Dylan McLeod on Unsplash. *A. clarkii* picture by Citron, and *C. viridis* photo by Ben Lancaster (both taken from Wikipedia)

Young adults (taken just after attaining adult size and sexual maturation, which is 2–3 years approx., for the *Amphiprion* species and 1 year for the damselfish) of *Amphiprion percula* (total length, 45.2 ± 1.2 mm; total weight, 1.6 ± 0.4 g; *n* = 12), *Amphiprion clarkii* (*L*_T_, 46.4 ± 5.1 mm; *W*_T_, 2.3 ± 1.19 g; *n* = 12) and *Chromis viridis* (*L*_T_, 43.0 ± 1.6 mm; *W*_T_, 1.3 ± 0.4 g; *n* = 12) (*Perciformes*, *Pomacentridae*) were used for the present study (Fig. 1). Fishes were acquired from local dealers and subjected to acclimation during 1 month in the facilities of the Marine Aquarium of the University of Murcia. Fish were kept in groups in a recirculating system (one tank per species, stock density of 0.30 g/L) under exactly the same conditions (temperature, 27 ± 1 °C; salinity, 24 ± 1; pH = 8 ± 0.2; dissolved oxygen, 6.5 ± 0.2 mg/L) and fed ad libitum twice a day a standard diet to match their requirements (Mysis shrimp, enriched *Artemia* nauplii and red plankton).

Fishes were euthanized by exposure to the anaesthetic tricaine methanesulfonate (MS222, 200 mg/L) for 10 min following the cessation of gill movement. Brain, livers and samples of skeletal muscle (collected above the lateral line, between the dorsal fin and the caudal fin) were collected, pooled and homogenized to produce quadruplicate samples (4 pools of 3 fish) for lipid analyses. Fresh samples were placed directly in chloroform/methanol (2:1, v/v) containing 0.01% (w/v) butylated hydroxytoluene as antioxidant and subjected to total lipid extraction (Folch et al. 1957).

### Lipid extraction and phospholipid class composition

Briefly, fish samples (2 g of skeletal muscle and 0.5 g of liver and brain) were homogenized in 20 mL of ice-cold chloroform/methanol followed by the addition of 5 mL of 0.88% (w/v) KCl, mixing, and layers allowed to separate on ice for 1 h. The upper aqueous layer was aspirated, and the lower organic layer was evaporated under a stream of oxygen-free nitrogen. All lipid extracts were stored at − 20 °C under a N_2_ atmosphere prior to analysis. PL classes were separated by high-performance thin-layer chromatography using 10- × 10-cm silica gel plates (VWR, Lutterworth, England) and methyl acetate/isopropanol/chloroform/methanol/0.25% (w/v) KCl (25:25:25:10:9, by volume) as solvent system (Olsen and Henderson 1989). The lipid classes were visualized by charring at 160 °C for 15 min after spraying with 3% (w/v) aqueous cupric acetate containing 8% (v/v) phosphoric acid and quantified by visible densitometry using Image Scanner II (Amersham Biosciences, UK). Scanned images were recorded automatically and analysed by computer using IQ-Image Quant TL 8.1 software (GE Healthcare Bio-Sciences AB, Sweden).

### Phospholipid fatty acid composition

Individual phospholipid (PL) classes from tissue’s total lipid extract were separated by preparative-TLC, using silica gel plates (20 × 20 cm) (VWR) and the solvent system as above. Individual PL classes were identified by comparison with known standards after spraying with 1% (w/v) 2′,7′-dichlorofluorescein in 97% (v/v) methanol containing 0.05% (w/v) BHT, and visualization under UV light. Each PL class was scraped from the plate into a test tube and subjected directly (on silica) to acid-catalysed transmethylation at 50 °C overnight following addition of 2 mL of 1% (v/v) sulphuric acid in methanol in order to prepare fatty acid methyl esters (FAME) (Christie 2003). FAME were separated and quantified by gas–liquid chromatography. For this, a Hewlett-Packard 5890 gas chromatograph with a capillary column (SPTH-2560, SUPELCO, 100 m × 0.25 mm I.D., 0.20-μm film thickness) was used. The oven temperature, held at an initial value of 140 °C for 5 min, was increased at a rate of 4 °C per min to 230 °C, then further increased at a rate of 1 °C per min to 240 °C and finally held at that temperature for 6 min. The injector and flame ionization detector were set at 250 °C. Helium at a pressure of 290 kPa was used as carrier gas. Peaks were identified by comparing their retention times with appropriate FAME standards purchased from Sigma Chemical Company (St. Louis, MO, USA). Individual fatty acid concentrations were expressed as percentages of the total content.

### Indexes and statistical analysis

The peroxidation index (PIn) was used as an estimate of every single PL susceptibility to oxidation and was calculated using the formula: PIn = 0.025 × (percentage of monoenoics) + 1 × (percentage of dienoics) + 2 × (percentage of trienoics) + 4 × (percentage of tetraenoics) + 6 × (percentage of pentaenoics) + 8 × (percentage of hexaenoics) (Witting and Horwitt 1964). PIn for total PL was calculated as the weighted sum of each PL’s PIn (Total PL’s PIn = p1PIn_PC_ + p2PIn_PE_ + p3PIn_CL_ + p4PIn_PS_ + p5PIn_PI_ + p6PIn_SM_, where p1–6 are the relative contents of each PL class). Results are presented as mean ± SD (*n* = 4). Where necessary, data were arc-sin transformed before further statistical analysis. A one-way analysis of variance (ANOVA) was used to compare individual PL proportions, single fatty acids or groups of fatty acids and indexes between tissues for each species and, then, to compare individual PL proportions, single fatty acids or groups of fatty acids and indexes between species for each tissue. Tukey’s post hoc test was used for multiple comparisons when pertinent, and data’s homogeneity of variances was checked by the Levene’s test. *P* < 0.05 was considered to be statistically different. Statistical analyses were performed using SPSS, version 22.0 (SPSS Inc., Chicago, IL).

## Results

### Phospholipid class composition

**Fig. 2:**
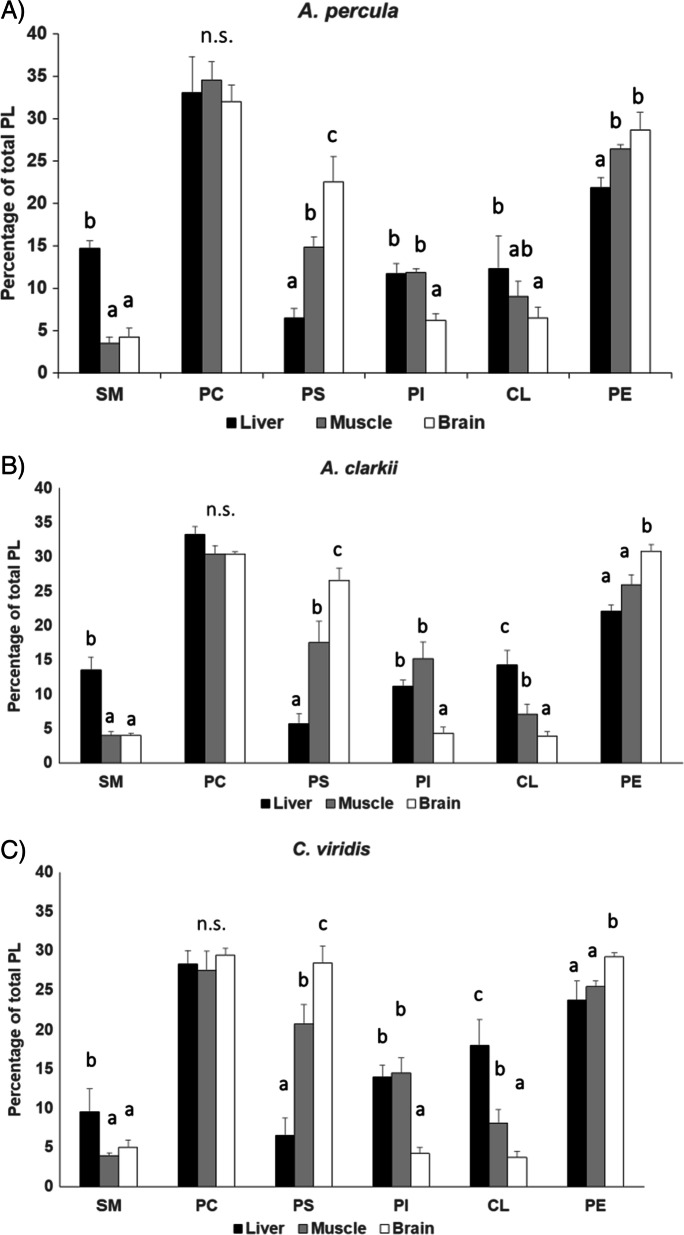
Phospholipid class composition (percentage of total phospholipids) of membranes isolated from three different tissues (liver, skeletal muscle and brain) of *Amphiprion percula* (**A**), *Amphiprion clarkii* (**B**) and *Chromis viridis* (**C**). Results are mean ± SD (*n* = 4). Superscript letters indicate the existence of statistical differences among tissues for each phospholipid class as determined by a one-way ANOVA and Tukey’s post hoc test (“b” indicates a statically higher value than “a” for the same PL class; *P* < 0.05). CL cardiolipin, n.s. non-significant, PC phosphatidylcholine, PE phosphatidylethanolamine, PI phosphatidylinositol, PS phosphatidylserine, SM sphingomyelin

**Fig. 3:**
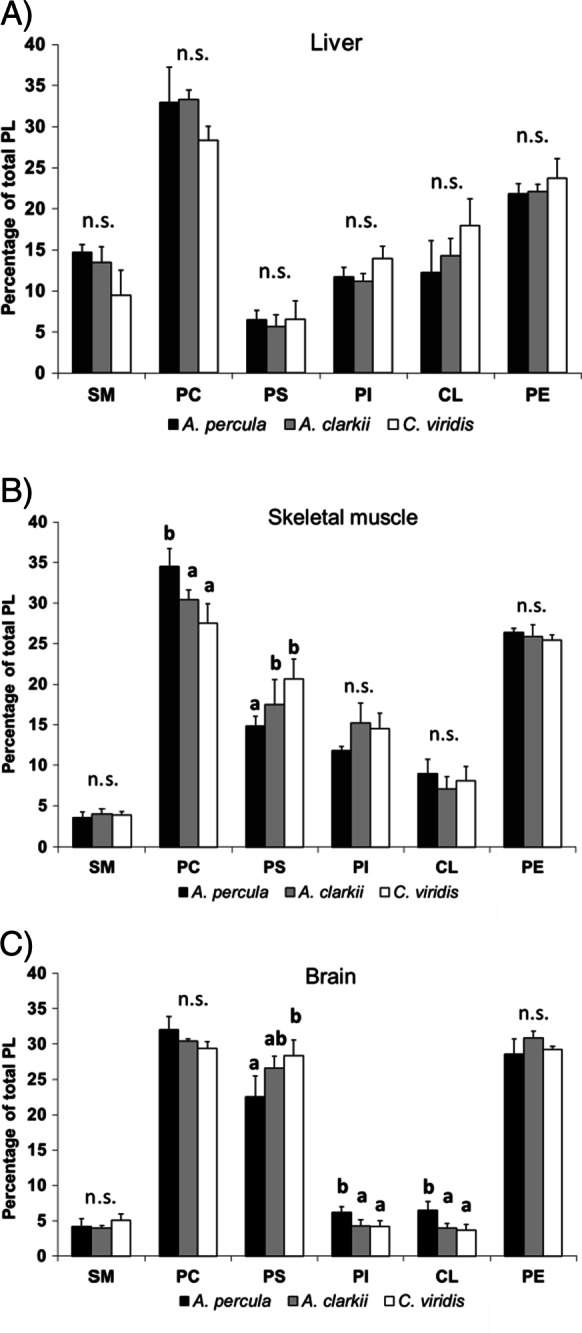
Phospholipid class composition (percentage of total phospholipids) of membranes isolated from liver (**A**), skeletal muscle (**B**) and brain (**C**) of young adult *Amphiprion percula*, *Amphiprion clarkii* and *Chromis viridis*. Results are mean ± SD (*n* = 4). Superscript letters indicate the existence of statistical differences among fish species for each phospholipid class as determined by a one-way ANOVA and Tukey’s post hoc test (“b” indicates a statically higher value than “a” for the same PL class; *P* < 0.05). CL cardiolipin, n.s. non-significant, PC phosphatidylcholine, PE phosphatidylethanolamine, PI phosphatidylinositol, PS phosphatidylserine, SM sphingomyelin

Percentages of the main phospholipid (PL) classes that integrate tissue membranes from *Amphiprion percula*, *A. clarkii* and *Chromis viridis* are represented in Fig. 2. Fish liver, skeletal muscle and brain showed different membrane PL compositions within each species. When the three tissues’ PL composition was compared for each fish species using a one-way ANOVA, liver membranes showed a significantly higher sphingomyelin (SM) relative content ([*F*(2,9 = 135.949, *P* < 0.001] for *A. percula*, [*F*(2,9) = 73.298, *P* < 0.001] for *A. clarkii* and [*F*(2,9) = 10.777, *P* = 0.005] for *C. viridis*) and a higher cardiolipin (CL) proportion than those from skeletal muscle and brain in the three species ([*F*(2,9 = 4.823, *P* = 0.048], [*F*(2,9) = 35.414, *P* < 0.001] and [*F*(2,9) = 43.775, *P* < 0.001] for *A. percula*, *A. clarkii* and *C. viridis*, respectively). Besides, liver membranes had a lower phoshatidylserine (PS) relative content ([*F*(2,9 = 52.876, *P* < 0.001], [*F*(2,9) = 34.824, *P* < 0.001] and [*F*(2,9) = 78.188, *P* < 0.001] for *A. percula*, *A. clarkii* and *C. viridis*, respectively). On the other hand, brain membranes showed higher PS and phosphatidylethanolamine (PE) ([*F*(2,9 = 16.222, *P* = 0.002], [*F*(2,9) = 41.046, *P* < 0.001] and [*F*(2,9) = 17.070, *P* = 0.001] for *A. percula*, *A. clarkii* and *C. viridis*, respectively) and lower phosphatidylinositol (PI) ([*F*(2,9 = 48.874, *P* < 0.001], [*F*(2,9) = 33.797, *P* < 0.001] and [*F*(2,9) = 57.707, *P* < 0.001] for *A. percula*, *A. clarkii* and *C. viridis*, respectively) and CL than those from liver and skeletal muscle in the three fish species. Regarding inter-species comparisons, no significant differences in PL class percentages were found in liver membranes among *Amphiprion percula*, *A. clarkii* and *Chromis clarkii* ([*F*(2,9) = 4.957, *P* = 0.054], [*F*(2,9) = 3.093, *P* = 0.119], [*F*(2,9) = 0.254, *P* = 0.784], [*F*(2,9) = 4.560, *P* = 0.062], [*F(*2,9) = 2.463, *P* = 0.166] and *F*(2,9) = 1.190, *P* = 0.367] for SM, PC, PI, PS, CL and PE, respectively) (Fig. 3A). Skeletal muscle from the coral reef damselfish *C. viridis* had higher relative contents of PS [F(2,9) = 11.390, *P* = 0.005] and lower of PC [*F*(2,9) = 10.753, *P* = 0.005] than *A. percula* (Fig. 3B), and brain membranes from *A. percula* showed a higher relative content in PI [*F*(2,9) = 7.304, *P* = 0.016] and CL [*F*(2,9) = 9.762, *P* = 0.007] and lower of PS [*F*(2,9) = 6.147, *P* = 0.024] than *C. viridis* (Fig. 3C).

### Fatty acid compositions and peroxidation index values of individual PLs

**Fig. 4:**
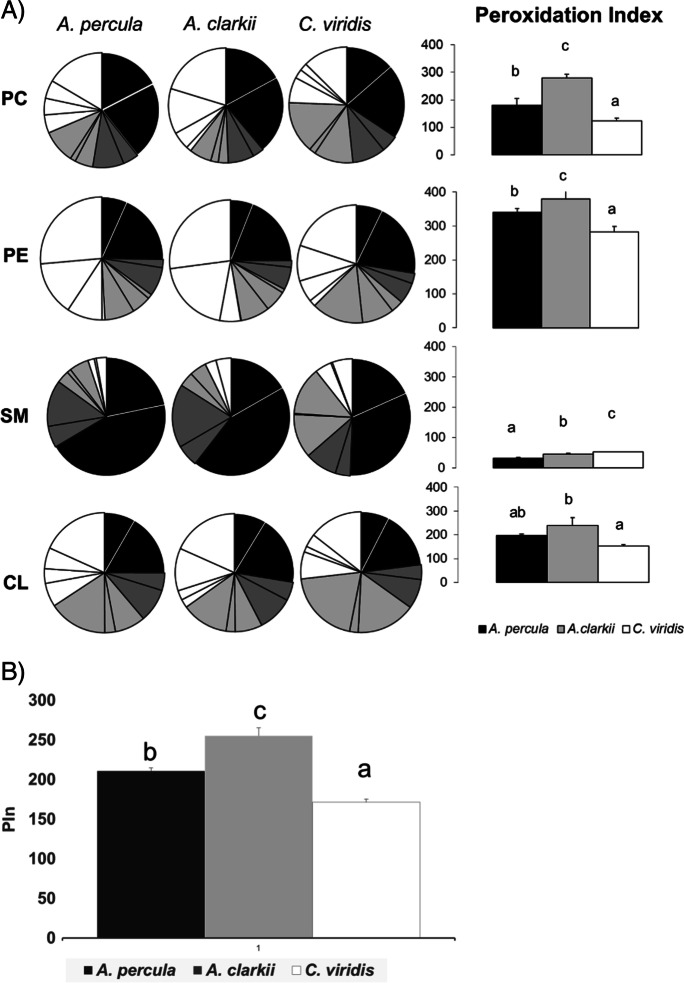
Phospholipid fatty acid composition of **liver** membranes from young adult *Amphiprion percula*, *Amphiprion clarkii* and *Chromis viridis*. Each segment of the pie chart represents the following fatty acids (clockwise order): saturated (black: 16:0 and Σsaturated), monounsaturated (dark grey: 18:1n-9 and Σmonounsaturated), n-6 polyunsaturated (light grey: 18:2, 20:4 and Σn-6 PUFA), n-3 polyunsaturated (white: 18:3, 20:5, 22:6 and Σn-3 PUFA). Right column graphs present peroxidation index (PIn) values of each PL class for the three fish species. **B**) PIn values for membrane total PL from liver of the three fish species. Results shown in PIn graphs are mean ± SD (*n* = 4). Superscript letters mean statistical differences among fish species for PIn values as determined by a one-way ANOVA and Tukey *t* test (“b” indicates a statically higher value than “a” for the same PL class; *P* < 0.05). CL cardiolipin, PC phosphatidylcholine, PE phosphatidylethanolamine, PI phosphatidylinositol, PS phosphatidylserine, SM sphingomyelin

**Fig. 5:**
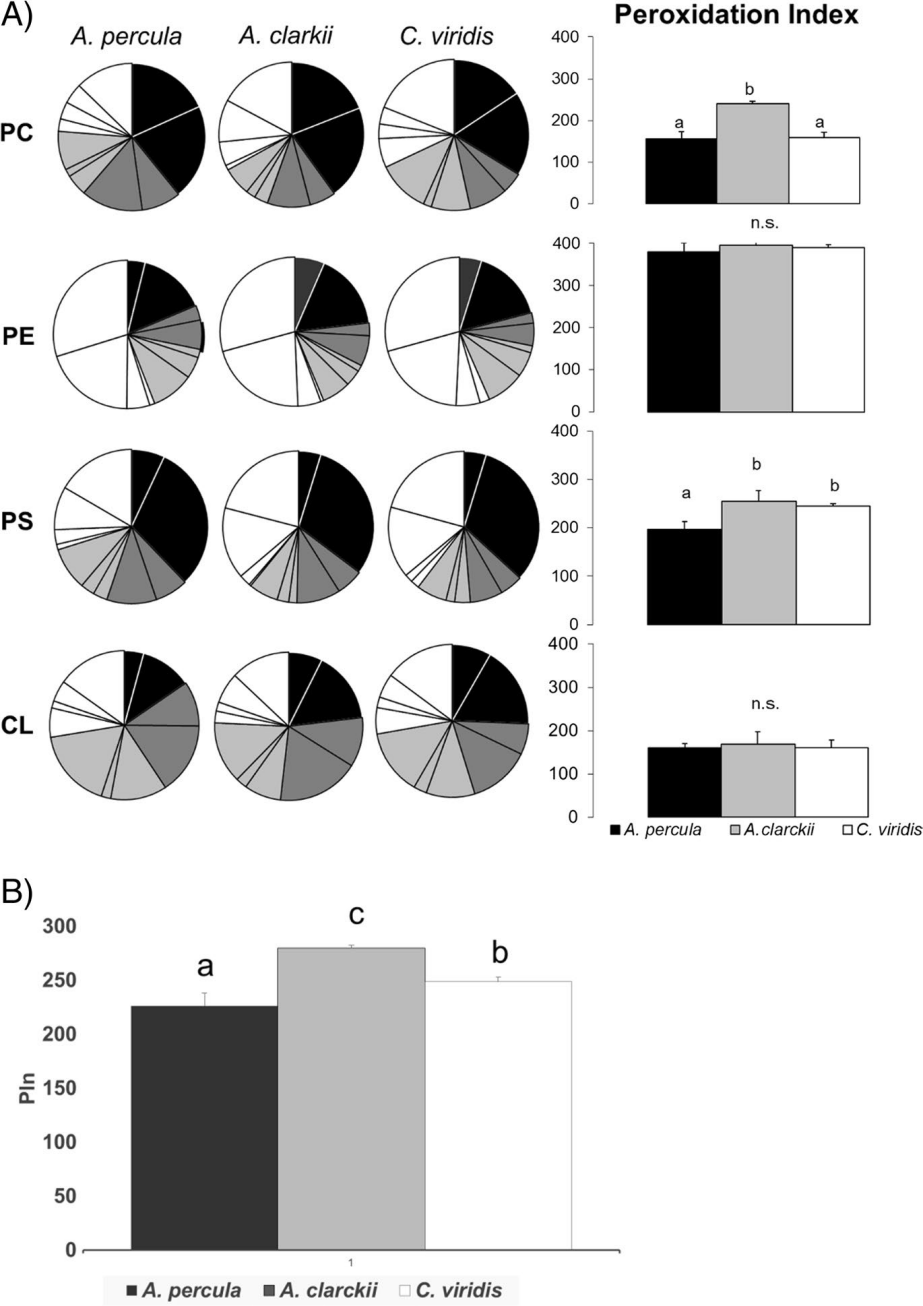
Phospholipid fatty acid composition of **skeletal muscle** membranes from young adult *Amphiprion percula*, *Amphiprion clarkii* and *Chromis viridis*. Each segment of the pie chart represents the following fatty acids (clockwise order): saturated (black: 16:0 and Σsaturated), monounsaturated (dark grey: 18:1n-9 and Σmonounsaturated), n-6 polyunsaturated (light grey: 18:2, 20:4 and Σn-6 PUFA), n-3 polyunsaturated (white: 18:3, 20:5, 22:6 and Σn-3 PUFA). Right column graphs present peroxidation index (PIn) values of each PL class for the three fish species. **B**) PIn values for membrane total PL from skeletal muscle of the three fish species. Results shown in PIn graphs are mean ± SD (*n* = 4). Superscript letters mean statistical differences among fish species for PIn values as determined by a one-way ANOVA and Tukey *t* test (“b” indicates a statically higher value than “a” for the same PL class; *P* < 0.05). CL cardiolipin, n.s. non-significant, PC phosphatidylcholine, PE phosphatidylethanolamine, PI phosphatidylinositol, PS phosphatidylserine, SM sphingomyelin

**Fig. 6:**
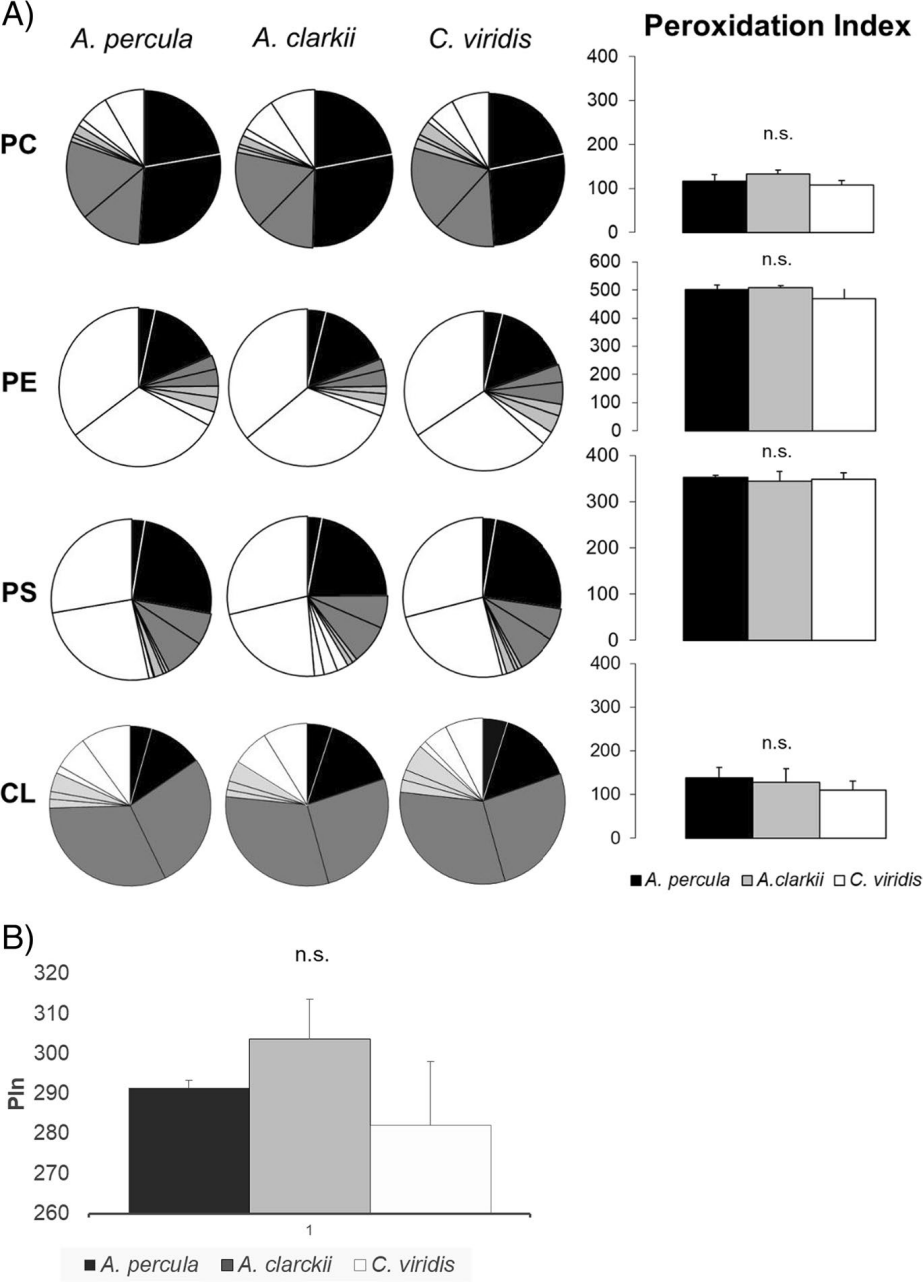
Phospholipid fatty acid composition of **brain** membranes from young adult *Amphiprion percula*, *Amphiprion clarkii* and *Chromis viridis*. Each segment of the pie chart represents the following fatty acids (clockwise order): saturated (black: 16:0 and Σsaturated), monounsaturated (dark grey: 18:1n-9 and Σmonounsaturated), n-6 polyunsaturated (light grey: 18:2, 20:4 and Σn-6 PUFA), n-3 polyunsaturated (white: 18:3, 20:5, 22:6 and Σn-3 PUFA). Right column graphs present peroxidation index (PIn) values of each PL class for the three fish species. **B**) PIn values for membrane total PL from brain of the three fish species. Results shown in PIn graphs are mean ± SD (*n* = 4). Superscript letters mean statistical differences among fish species for PIn values as determined by a one-way ANOVA and Tukey *t* test (“b” indicates a statically higher value than “a” for the same PL class; *P* < 0.05). CL cardiolipin, n.s. non-significant, PC phosphatidylcholine, PE phosphatidylethanolamine, PI phosphatidylinositol, PS phosphatidylserine, SM sphingomyelin

Figures 4, 5 and 6 show the fatty acid composition and peroxidation index (PIn) values of tissue membranes (liver, skeletal muscle and brain, respectively) from young adult *Amphiprion percula*, *Amphiprion clarkii* and *Chromis viridis* kept under equal temperature and rearing conditions and fed the same diet. For clarity reasons, pie charts in every figure represent only the groups of fatty acids (sum of saturated, sum of monounsaturated, sum of n-6 polyunsaturated and sum of n-3 polyunsaturated) and the most representative fatty acids within each group. Only the four more significant PL for each tissue (the three more abundant PL plus CL, the sum of which was at least 81.8% of total PL) are presented. The complete fatty acid composition and indexes for every PL class and tissue for the three experimental fish species are included as Supplementary material (Supp. Tables 1–18).

Regarding fish liver, the peroxidation index (PIn) was significantly lower in *C. viridis* than in the two Amphiprion species for all PL classes ([*F*(2,9) = 23.775, *P* = 0.001], [*F*(2,9) = 12.920, *P* = 0.004], [*F*(2,9) = 9.670, *P* = 0.01], [*F*(2,9) = 11.652, *P* = 0.009], [*F*(2,9) = 71.658. *P* < 0.001] for PE, CL, PI, PS and PC, respectively), except for sphingomyelin (SM) [*F*(2,9) = 40.068, *P* = 0.001], in which PIn was higher in *C. viridis* (PIn = 52.1 ± 1.3) than in the other two fish species (PIn = 31.7 ± 2.5 and 45.2 ± 3.2 for *A. percula* and *A. clarkii*, respectively) (Fig. 4, Supp. Tables 1–6). PIn values for *A. clarkii* were higher than those for *A. percula* for PC (279.4 ± 13.5 vs. 180.3 ± 24.1), PE (380.7 ± 22.6 vs. 340.7 ± 11.3) and SM. PIn value for total PL was significantly lower in *C. viridis* (171.5 ± 3.7) [*F*(2,9) = 126.387, *P* < 0.001]. Regarding the two *Amphiprion* species, PIn of total PL was higher in *A. clarkii* (255.4 ± 9.7) than in *A. percula* (210.9 ± 4.0). Liver membranes from *C. viridis* showed a lower content in n-3 polyunsaturated fatty acids (PUFA) in PC (22.0 ± 1.2), PE (33.0 ± 1.5) and PS (26.0 ± 1.6) and higher in SM (9.3 ± 0.8) when compared with *A. percula* (29.1 ± 4.0, 43.7 ± 1.1, 35.8 ± 1.9 and 4.3 ± 1.3 for PC, PE, PS and SM, respectively) and *A. clarkii* (36.4 ± 1.8, 45.3 ± 6.7, 37.3 ± 4.9 and 6.0 ± 2.4) ([*F*(2,9) = 24.168, *P* < 0.001] for PC, [*F*(2,9) = 8.154, *P* = 0.019] for PE, [*F*(2,9) = 11.418, *P* = 0.009] for PS and [*F*(2,9) = 6.763, *P* = 0.045] for SM).

There were significant differences in PIn values for skeletal muscle membranes among the experimental fish species for PC [*F*(2,9) = 43.240, *P* < 0.001], PS [*F*(2,9) = 12.086, *P* = 0.004] and PI [*F*(2,9) = 50.998, *P* < 0.001] (Fig. 5, Supp. Tables 7–12). PIn values for PS and PI were lower in *A. percula* (196.5 ± 16.9 and 242.1 ± 7.5) than in *A. clarkii* (255.1 ± 21.9 and 294.0 ± 6.2) and *C. viridis* (244.4 ± 6.1 and 282.6 ± 7.2), while PC PIn was higher in *A. clarkii* (240.4 ± 5.1) compared to *A. percula* (155.9 ± 16.8) and *C. viridis* (158.5 ± 13.6). Regarding the two *Amphiprion* species, PIn values for PC, PS and PI were lower in *A. percula* compared to *A. clarkii*. Total PL PIn value was significantly higher in *A. clarkii* (279.7 ± 2.7) than in *C. viridis* (248.8 ± 4.0) and *A. percula* (226.5 ± 11.4) [*F*(2,9) = 46.666, *P* < 0.001]. Skeletal muscle membranes from *A. percula* showed a lower n-3 PUFA content in PC (22.8 ± 1.9), PS (24.8 ± 1.9) and PI (22.2 ± 1.4) than *A. clarkii* (31.8 ± 0.3, 31.2 ± 4.0 and 30.5 ± 1.8) and *C. viridis* (26.9 ± 2.1, 31.3 ± 0.8 and 36.0 ± 1.3) ([*F*(2,9) = 19.707, *P* < 0.001] for PC, [*F*(2,9) = 6.497, *P* = 0.021] for PS and [*F*(2,9) = 70.368, *P* < 0.001]).

Regarding fish brain, PIn values showed no significant differences among fish species for any of the four most significant PL classes or for total PL (Fig. 6, Supp. Tables 13–18).

## Discussion

Membrane lipid composition significantly differed among *Amphiprion percula, A. clarkii* and *Chromis viridis* in the three analysed tissues but the direction and magnitude of the observed differences did not always explain the existing divergence in the estimated maximum lifespan potential (MLSP) among the species. Membranes from each tissue had distinctive phospholipid (PL) proportions and PL fatty acid compositions, which is in alignment with previous data in fish (Almaida-Pagán et al. 2012a, b) and rats (Paradies et al. 1992; Modi et al. 2008) and is very likely related to differential functional properties of membranes in each tissue, so as to different susceptibilities to lipid peroxidation. While liver membranes were richer in sphingomyelin (SM) and cardiolipin (CL), those from brain had a higher content in phophatidylserine (PS) and phosphatidylethanolamine (PE) than the other tissues. As discussed below, a high PE content may play a protective role from the ageing-associated damage caused by ROS in this tissue in particular (Feng et al. 2014). This is interesting as brain had the most unsaturated membranes compared to liver and skeletal muscle within each species, which render them more sensitive to oxidative damage (Hulbert et al. 2006b).

Tissue membranes showed significant differences in PL proportions among the three species. PS content was statistically higher in skeletal muscle and brain from the shorter-lived *C. viridis* (estimated MLSP = 1–2 years) compared to the two *Amphiprion* species (estimated MLSP of 30 and 9–16 for *A. percula* and *A. clarkii*, respectively). This is in accordance with that observed in a previous study where three fish species of the short-lived annual genus *Nothobranchius* (*Nothobranchius korthausae*, *Nothobranchius rachovii*, *Nothobranchius guentheri*) with different MLSP (MLSP = 80, 63 and 53 weeks, respectively) and the longer-lived outgroup species *Aphyosemion australe* (MLSP = 156 weeks) were studied to test whether they conform to the predictions of the LHA theory of ageing (de Costa et al. 2020). A negative correlation between fish MLSP and PS content from cell membranes was also found in the *Nothobranchius* study and suggested to be linked to PS decarboxylation. PS descarboxylation leads to an increase in phosphatidylethanolamine (PE) intracellular levels, which was also found in the longer-lived *Nothobranchius* species and in *A. australe*. Since the abundance of PE positively regulates autophagy, regarded as one of the major cytoprotective mechanisms during ageing (Feng et al. 2014), both a lower content of PS and higher of PE in cell membranes could indicate that this mechanism is operating to protect cells and tissues from the ageing-associated damage caused by ROS (de Costa et al. 2020). Nevertheless, no statistical differences among *Amphiprion* species and *C. viridis* in PE levels were found in any tissue’s membranes.

Regarding membrane PL fatty acid composition of fish tissues from the two *Amphiprion* species, *A. percula* had less unsaturated membranes and, thus, lower peroxidation levels in liver and skeletal muscle (not statistical differences were found for brain) than *A. clarkii*, which has an estimated MLSP of half that of *A. percula* (9–16 vs. 30 years). This occurred at the level of the main PL classes from the two tissues, as it was shown in whole body of the *Nothobranchius* species previously studied (de Costa et al. 2020) and supports the longevity-homeovicous adaptation (LHA) theory of ageing (Pamplona et al. 1998, 2000, 2002; Naudí et al. 2013). In previous studies performed by Hulbert et al., a strong inverse relationship was found between peroxidation index (PIn) of liver mitochondrial PLs and skeletal muscle PLs and MLSP of mammals (Hulbert 2005; Hulbert et al. 2006a) (see Fig. 7 in Hulbert et al., 2007). The relationship between liver mitochondrial phospholipid PIn of mammals is proportional to their MLSP^−0.40^, which means that a 24% decrease in their peroxidative susceptibility is associated with every doubling of MSLP. For skeletal muscle membranes, the corresponding value is that a 19% decrease in peroxidative susceptibility is associated with every doubling of MLSP in mammals (muscle PIn is proportional to MLSP^−0.30^). Our data reflected a PIn reduction of 17.4% and 19% in liver and skeletal muscle membrane phospholipids, respectively, which is quite close to that observed in mammals.

When membrane PL fatty acid composition from tissues of the two *Amphiprion* species was compared with that of *C. viridis*, we found that *C. viridis* membranes had generally a lower PIn value than that from one (in skeletal muscle) or the two *Amphiprion* species (in liver), this being in contradiction with the LHA theory of ageing. This is not the first time that we obtain data that apparently contradict the theory. In a previous study, we compared mitochondrial membrane lipids from whole *Nothobranchius rachovii* (MLSP = 14 months) and *Nothobranchius furzeri* (MLSP = 7 months), which resulted from two separate experiments (Lucas-Sanchez et al. 2014; Almaida-Pagán et al. 2019), and showed that the shorter-lived species had the lowest PIn values. Afterwards, lipid profiles from whole *N. korthausae*, *N. rachovii*, *N. guentheri* and *Aphyosemion australe* kept under the same feeding and housing conditions were correlated with the MLSP of each species (MLSP = 80, 63, 53 and 156 weeks, respectively) (de Costa et al. 2020). Results showed a negative correlation between membranes total PIn and fish MLSP, meaning that the most long-lived *Nothobranchius* species have a lower susceptibility to oxidative damage, which was in accordance with the LHA theory of ageing. However, the magnitude of the observed decrease in PIn associated with every doubling of MLSP was of only 2%, which may indicate that specific tissues contribute more than others to this relationship, the inter-tissue differences masking the overall PIn when the whole fish is analysed.

In the present study and the case of *C. viridis*, *Chromis* fishes are considered a priori a model for short-lived reef inhabitants (Wantiez and Thollot P. 2001). Although they belong to a different genus, *C. viridis* share a similar habitat and feeding behaviour with clownfish. The main difference between them is that *C. viridis* undergo severe predation in the post-settlement phase (Hixon and Carr 1997) and have high juvenile and adult mortality. This, combined with a very rapid growth (80% of maximum size reached within the first year), clearly indicates that these animals are short-lived in the wild, and therefore, tissues with more unsaturated membranes should be expected in order to cope with the LHA theory of ageing.

In conclusion, the present study showed differences in membrane composition (phospholipid class and fatty acid compositions) among fish tissues that point to the importance of particular PLs for tissue-specific functions. Significant differences in liver, skeletal muscle and brain membranes among *A. percula*, *A. clarkii* and *C. viridis* were found. When only the two *Amphiprion* species were compared, results pointed to the existence of a negative relationship between membrane PIn value and lifespan, as it has previously been shown in mammals, birds and fish species of genus *Nothobranchius*. Nevertheless, when the two *Amphiprion* species were compared to the shorter-lived *C. viridis*, data contradicted what the LHA theory of ageing posits. Although new studies including a wider number of anemonefish and other phylogenetically related species with different MLSP should be carried out to reinforce what was found in the present work, this data along with those obtained in previous studies on fish denote that the magnitude (and sometimes the direction) of the differences observed in membrane lipid composition and peroxidation index with maximum lifespan cannot explain alone the diversity in longevity found among fishes.

## Supplementary Information

Below is the link to the electronic supplementary material.

Supplementary file1 (RAR 210 KB)

## Data Availability

The datasets generated during and/or analysed during the current study are available from the corresponding author on reasonable request.

## Abbreviations

BHT: Butylated hydroxytoluene
CL: Cardiolipin
FAME: Fatty acid methyl esters
LHA: Longevity-homeoviscous adaptation theory of ageing
mtROSp: Mitochondrial ROS production
MLSP: Maximum lifespan potential
PC: Phosphatidylcholine
PE: Phosphatidylethanolamine
PI: Phosphatidylinositol
PIn: Peroxidation index
PL: Phospholipids
PS: Phosphatidylserine
PUFA: Polyunsaturated fatty acids
ROS: Reactive oxygen species
SM: Sphingomyelin
TLC: Thin layer chromatography
